# Growth inhibition of pathogenic microorganisms by *Pseudomonas protegens* EMM-1 and partial characterization of inhibitory substances

**DOI:** 10.1371/journal.pone.0240545

**Published:** 2020-10-15

**Authors:** Catherine Cesa-Luna, Antonino Baez, Alberto Aguayo-Acosta, Roberto Carlos Llano-Villarreal, Víctor Rivelino Juárez-González, Paul Gaytán, María del Rocío Bustillos-Cristales, América Rivera-Urbalejo, Jesús Muñoz-Rojas, Verónica Quintero-Hernández

**Affiliations:** 1 Ecology and Survival of Microorganisms Group (ESMG), Laboratorio de Ecología Molecular Microbiana (LEMM), Centro de Investigaciones en Ciencias Microbiológicas (CICM), Instituto de Ciencias (IC), Benemérita Universidad Autónoma de Puebla (BUAP), Puebla, Pue., México; 2 Department of Microbiology and Immunology, Biological Sciences Faculty, Universidad Autónoma de Nuevo León, Ciudad Universitaria, San Nicolás de la Garza, Nuevo León, México; 3 Departamento de Medicina Molecular y Bioprocesos, Instituto de Biotecnología, Universidad Nacional Autónoma de México, Cuernavaca, Morelos, México; 4 Unidad de Síntesis y Secuenciación de ADN, Instituto de Biotecnología, Universidad Nacional Autónoma de México, Cuernavaca, Morelos, México; 5 Facultad de Estomatología, BUAP, Puebla, Pue., México; 6 CONACYT–ESMG, LEMM, CICM, IC-BUAP, Puebla, Pue., México; Defense Threat Reduction Agency, UNITED STATES

## Abstract

The bacterial strain, EMM-1, was isolated from the rhizosphere of red maize (“Rojo Criollo”) and identified as *Pseudomonas protegens* EMM-1 based on phylogenetic analysis of 16S rDNA, *rpoB*, *rpoD*, and *gyrB* gene sequences. We uncovered genes involved in the production of antimicrobial compounds like 2,4-diacetylphloroglucinol (2,4-DAPG), pyoluteorin, and lectin-like bacteriocins. These antimicrobial compounds are also produced by other fluorescent pseudomonads alike *P*. *protegens*. Double-layer agar assay showed that *P*. *protegens* EMM-1 inhibited the growth of several multidrug-resistant (MDR) bacteria, especially clinical isolates of the genera *Klebsiella* and β-hemolytic *Streptococcus*. This strain also displayed inhibitory effects against diverse fungi, such as *Aspergillus*, *Botrytis*, and *Fusarium*. Besides, a crude extract of inhibitory substances secreted into agar was obtained after the cold-leaching process, and physicochemical characterization was performed. The partially purified inhibitory substances produced by *P*. *protegens* EMM-1 inhibited the growth of *Streptococcus* sp. and *Microbacterium* sp., but no inhibitory effect was noted for other bacterial or fungal strains. The molecular weight determined after ultrafiltration was between 3 and 10 kDa. The inhibitory activity was thermally stable up to 60°C (but completely lost at 100°C), and the inhibitory activity remained active in a wide pH range (from 3 to 9). After treatment with a protease from *Bacillus licheniformis*, the inhibitory activity was decreased by 90%, suggesting the presence of proteic natural compounds. All these findings suggested that *P*. *protegens* EMM-1 is a potential source of antimicrobials to be used against pathogens for humans and plants.

## Introduction

Microorganisms have developed diverse strategies to survive and compete for the resources of their habitat, and one of them is the production of inhibitory substances [1]. Pseudomonads are Gram-negative γ-proteobacteria, highly competitive due to the production of several inhibitory compounds [2]. Pseudomonads can be used for biocontrol due to broad-spectrum antibiotics, bacteriocins, and siderophores production [3–5]. *Pseudomonas protegens* represents a new bacterial group that stands out for its ability to produce diverse inhibitory substances, including pyoluteorin (Plt), pyrrolnitrin (Prn), 2,4-diacetylphloroglucinol (2,4-DAPG), and hydrogen cyanide [6, 7]. Some of the most studied strains are *P*. *protegens* Pf-5, CHA0, and Cab57 for contributing to plant protection [8]. Besides producing antifungal compounds, *P*. *protegens* produces LlpA bacteriocins [9], capable of inhibiting some phytopathogenic *Pseudomonas*. These bacteriocins likely provide *P*. *protegens* with competitive advantages in the rhizosphere [2].

Although the commercial use of inhibitory substances, such as bacteriocins, is limited, they have been considered as promising weapons against diverse pathogens because they have been used for years as natural food preservatives [10]. At the same time, the success obtained in the elimination of pathogens through *in vivo* experiments has led the consideration of their clinical use to treat different bacterial infections. For example, amyloliquecidine (produced by *Bacillus amyloliquefaciens*) and pyocin SD2 (from *P*. *aeruginosa*) are effective in treating skin and lung infections in animal models, respectively [11, 12].

Antimicrobial resistance (AMR) is an emerging problem that conditions the greater use of broad-spectrum antibiotics, causing negative consequences in the health, environment, and agriculture sectors [13]. Moreover, the presence of resistant bacteria increases the risk of spreading and prolonging infectious diseases. In early 2017, the World Health Organization (WHO) published a list of pathogenic bacteria for which new antibiotics are needed. The list included *Enterococcus* spp., *Staphylococcus aureus*, *Klebsiella pneumoniae*, *Acinetobacter baumannii*, *P*. *aeruginosa*, and *Enterobacter* spp. [14]. In Mexico, AMR has increased significantly in recent years for these pathogens. For example, up to 62% of *S*. *aureus* isolates are resistant to methicillin. The frequency of resistance of *Klebsiella* strains to β-lactam and carbapenem antibiotics is 56 and 4.6%, respectively. *K*. *pneumoniae* is resistant to third-generation cephalosporins and carbapenems [15]. Another microorganism of clinical importance is *Mycobacterium tuberculosis*. Approximately 1.5 million people die per year due to the increase of multidrug-resistant (MDR) strains. In 2015, 10.4 million new cases of tuberculosis were registered worldwide, and 580,000 cases corresponded to MDR tuberculosis [16]. In 2016, patients with extensively drug-resistant tuberculosis (XDR-TB) were reported worldwide.

Antimicrobial resistance has also affected other areas, such as agriculture, and the indiscriminate use of agrochemicals has affected human health [17]. For example, resistance to streptomycin (an antibiotic used to control bacterial diseases in crops) has been reported in plant pathogens, such as *P*. *syringae* and *Xanthomonas campestris* [18]. The increase of fungicide-resistant fungi has also been reported, reducing the effectiveness of control agents, which hinders the agronomic management of diseases in crops [19].

Currently, the study of inhibitory substances produced by antagonistic bacteria is gaining importance in developing alternative solutions for food preservation and probiotic therapeutics as well as antibacterial agents against MDR pathogens or to control fungal diseases in crop plants [20]. In this regard, the study of inhibitory substances from rhizospheric bacteria is valuable for the development of new antimicrobials to combat “super bacteria” or to develop new biocontrol strategies.

In this work, we report the identification of *P*. *protegens* EMM-1, a strain isolated from the “Rojo Criollo” maize rhizosphere. The molecular identification was performed by amplification, sequencing, and phylogenetic analysis of the 16S rDNA gene and three housekeeping genes (*rpoB*, *rpoD*, and *gyrB*). Amplification of genes related to the production of antibiotics in *P*. *protegens* species was also included.

Our results demonstrated that *P*. *protegens* EMM-1 inhibited several Gram-positive and Gram-negative bacteria (including MDR clinical isolates) as well as some important fungi. Additionally, the physicochemical characterization of partially purified inhibitory substances produced by *P*. *protegens* EMM-1 in the solid medium was performed. These findings suggested a promising use of *P*. *protegens* EMM-1 as a source of antimicrobials with the potential to be used against human or plant pathogens.

## Materials and methods

### Bacterial and fungal strains

The bacterial strain EMM-1 was isolated in 2008 from the rhizosphere of the autochthonous red maize (named “Rojo Criollo”) at the Ecology and Survival of Microorganisms Group from the Benemérita Universidad Autónoma de Puebla at Puebla, Mexico. “Rojo Criollo” maize was extracted from rhizospheric soil (19° 10’ 30.59” N, 98° 09’ 50.05” W, Elevation: 2408 MASL) [21]. Several strains were isolated, for example, *Enterobacter* sp. UAPS03001 [21] and others that are not characterized yet. EMM-1 was first grown on Congo Red agar [22] and subsequently in the LB medium. The EMM-1 strain was selected due to its inhibitory activity against some bacteria isolated from the same samples. EMM-1 was preliminarily identified as *Pseudomonas* sp. EMM-1 based on microscopic observation of Gram-negative bacilli, after Gram staining, and amplification of the 16S rDNA gene sequence (GenBank accession number: MN959751.1).

To study the range of inhibition of EMM-1 strain, the inhibitory activity was tested against several bacterial and fungal strains (Table 1). We selected a total of 32 target strains as follows: 15 MDR clinical isolates from patients with respiratory infectious diseases at ISSSTEP (Instituto de Seguridad y Servicios Sociales de los Trabajadores al Servicio de los Poderes del Estado de Puebla); nine bacterial strains isolated from the rhizosphere, roots, or the surface of diverse plants previously described (Table 1); four strains from the American Type Culture Collection (ATCC; Manassas, VA, USA); and four fungal strains formerly isolated from infected plants and identified based on their macro/microscopic characteristics (data not shown).

**Table 1:** Bacterial and fungal strains used in this work.

Microorganism	Isolation and relevant features	Culture medium and growth conditions	Reference
**Bacterial strains**			
1. *Burkholderia cepacia*	Isolated from sugarcane. Opportunistic pathogen. MDR.	BAc^a^, 30°C, 24 h.	[23]
2. *Burkholderia cenocepacia*	Isolated from sugarcane. Opportunistic pathogen. MDR.	BAc, 30°C, 24 h.	[23]
3. *Burkholderia multivorans*	Isolated from sugarcane. Opportunistic pathogen. MDR.	BAc, 30°C, 24 h.	[23]
4. *Burkholderia dolosa*	Isolated from sugarcane. Opportunistic pathogen. MDR.	BAc, 30°C, 24 h.	[23]
5. *Escherichia coli* ATCC 25922	Type strain.	MacConkey, 30°C, 24 h.	ATCC
6. *Klebsiella pneumoniae* subsp. *pneumoniae* ATCC 13883	Type strain. Opportunistic pathogen.	MacConkey, 30°C, 24 h.	ATCC
7. *Klebsiella* sp. KP1	Isolated from pharyngeal exudates. ISSSTEP hospital, Puebla, Mexico. MDR.	MacConkey, 30°C, 24 h.	This work
8. *Klebsiella* sp. KP2	Isolated from pharyngeal exudates. ISSSTEP hospital, Puebla, Mexico. MDR.	MacConkey, 30°C, 24 h.	This work
9. *Klebsiella* sp. KP3	Isolated from pharyngeal exudates. ISSSTEP hospital, Puebla, Mexico. MDR.	MacConkey, 30°C, 24 h.	This work
10. *Klebsiella* sp. KP4	Isolated from pharyngeal exudates. ISSSTEP hospital, Puebla, Mexico. MDR.	MacConkey, 30°C, 24 h.	This work
11. *Klebsiella* sp. KP6	Isolated from vaginal exudates. ISSSTEP hospital, Puebla, Mexico. MDR.	MacConkey, 30°C, 24 h.	This work
12. *Klebsiella* sp. KP7	Isolated from urine. ISSSTEP hospital, Puebla, Mexico. MDR.	MacConkey, 30°C, 24 h.	This work
13. *Klebsiella* sp. KP10	Isolated from urine. ISSSTEP hospital, Puebla, Mexico. MDR.	MacConkey, 30°C, 24 h.	This work
14. *Klebsiella* sp. KP12	Isolated from urine. ISSSTEP hospital, Puebla, Mexico. MDR.	MacConkey, 30°C, 24 h.	This work
15. *Klebsiella* sp. KP17	Isolated from urine. ISSSTEP hospital, Puebla, Mexico. MDR.	MacConkey, 30°C, 24 h.	This work
16. *Klebsiella variicola* T29A	Isolated from sugarcane. Plant-growth promoter. Tolerant to desiccation.	MacConkey, 30°C, 24 h.	[24]
17. *Microbacterium* sp. UAPS01-201	Isolated from leaf surface of maize. Sensitive to the inhibitory activity of EMM-1 strain.	LB^b^, 30°C, 24–48 h.	This work
18. *Paraburkholderia tropica* MTo-293	Isolated from maize rhizosphere. Isolated from maize rhizosphere. N2-fixing. Biocontrol.	BAc, 30°C, 24 h.	[25]
19. *Paraburkholderia unamae* MTl-641^T^	Isolated from maize rhizosphere. N2-fixing and endophytic specie.	BAc, 30°C, 24 h.	[26]
20. *Paraburkholderia unamae* SCCu-23	Isolated from sugarcane roots.	BAc, 30°C, 24 h.	[27]
21. *Raoultella planticola* ATCC 33531	Type strain. Isolated from radish root.	MacConkey, 30°C, 24 h.	ATCC
22. *Staphylococcus aureus* subsp. *aureus* ATCC 25923	Type strain. Opportunistic pathogen.	MSA^c^, 30°C, 24 h.	ATCC
23. *Streptococcus* sp. SP9	Isolated from pharyngeal exudates. ISSSTEP hospital, Puebla, Mexico. β-hemolytic. MDR.	BA^d^, 30°C, 24–48 h.	This work
24. *Streptococcus* sp. SP10	Isolated from pharyngeal exudates. ISSSTEP hospital, Puebla, Mexico. β-hemolytic. MDR.	BA, 30°C, 24–48 h.	[28]
25. *Streptococcus* sp. SP13	Isolated from pharyngeal exudates. ISSSTEP hospital, Puebla, Mexico. β-hemolytic. MDR.	BA, 30°C, 24–48 h.	This work
26. *Streptococcus* sp. SP14	Isolated from pharyngeal exudates. ISSSTEP hospital, Puebla, Mexico. β-hemolytic. MDR.	BA, 30°C, 24–48 h.	This work
27. *Streptococcus* sp. SP17	Isolated from pharyngeal exudates. ISSSTEP hospital, Puebla, Mexico. β-hemolytic. MDR.	BA, 30°C, 24–48 h.	This work
28. *Streptococcus* sp. SP20	Isolated from pharyngeal exudates. ISSSTEP hospital, Puebla, Mexico. β-hemolytic. MDR.	BA, 30°C, 24–48 h.	This work
**Fungal strains**			
29. *Aspergillus* sp.	Isolated from red maize.	PDA^e^, 30°C, 120 h	This work
30. *Botrytis* sp.	Isolated from vineyard soil, Atlixco, Puebla, Mexico.	PDA, 30°C, 120 h	This work
31. *Fusarium* sp.	Isolated from red maize.	PDA, 30°C, 120 h	This work
32. *Rhizopus* sp.	Isolated from vineyard soil, Atlixco, Puebla, Mexico.	PDA, 30°C, 120 h	This work

^a^Burkholderia Azelaic citrulline

^b^Luria-Bertani

^c^Mannitol Salt Agar

^d^Blood agar

^e^Potato Dextrose agar.

Microbial cultures were grown at 30°C and preserved in 50% glycerol at -80°C to provide stable inoculums during the study. Growth conditions and culture media used for each microorganism are specified in Table 1.

### Bacterial DNA extraction, amplification, DNA sequencing and phylogenetic analysis

For bacterial identification, genomic DNA from the EMM-1 strain was extracted using the Promega Wizard® Genomic DNA Purification Kit (Promega, Madison, WI, USA) according to the manufacturer’s instructions. The 16S rDNA gene and three housekeeping genes (*rpoB*, *rpoD*, and *gyrB*) were amplified by PCR using the primers listed in the S1 Table.

PCR assays were performed in a 25-μl reaction volume using GoTaq® Green Master Mix (Promega, Madison, WI, USA) with the following amplification conditions: 1) amplification of 16S rDNA consisted of denaturation at 95°C for 5 min followed by 30 cycles of denaturation at 95°C for 30 sec, annealing at 52°C for 40 sec, and extension at 72°C for 1 min with a final extension at 72°C for 5 min; 2) amplification of *rpoB*, *rpoD*, and *gyrB* consisted of denaturation at 94°C for 2 min followed by 30 cycles of denaturation at 94°C for 1 min, annealing at the corresponding Tm (S1 Table) for 45 sec, and extension at 72°C for 30 sec with a final extension at 72°C for 10 min.

PCR amplifications were verified by agarose gel electrophoresis, and the amplified genes were purified using the QIAquick Gel Extraction Kit (Qiagen, Hilden, Germany) according to the manufacturer’s instructions. The amplified genes were sequenced by Unidad de Síntesis y Secuenciación de ADN (IBt-UNAM, Mexico). Nucleotide sequences were analyzed using BlastN [29] against the GenBank nucleotide database.

Phylogenetic analysis of partial 16S rDNA gene sequences was carried out using EMM-1 strain and 24 strains belonging to the *P*. *fluorescens* group retrieved from GenBank. Sequence alignment was performed using the Clustal W program (University College Dublin, Ireland) [30] and corrected in BioEdit (Carlsbad, CA, USA) [31]. A neighbor-joining [32] tree was inferred from evolutionary distances calculated with the Kimura 2-parameter method in MEGA X version 10.1.7 (Philadelphia, PA, USA) [33], and confidence analysis was undertaken with 1000 bootstrap replicates. Phylogenetic analysis was also performed on individual housekeeping genes after concatenation of 16S rDNA, *rpoB*, *rpoD*, and *gyrB*. This analysis was carried out with the same strains used for 16S rDNA gene phylogeny, using nucleotide sequences available from GenBank, and following the same methodology.

### Amplification of genes required for the synthesis of antimicrobial metabolites

*P*. *protegens* synthesize the antimicrobial metabolites, 2,4-DAPG, Plt, and LlpA which require the *phlD*, *plt*, and *llpA* genes, respectively [2, 4, 9]. Therefore, we amplified and sequenced these genes to further determine if EMM-1 strain has the potential to synthesize these particular antimicrobials. For amplification, we designed the catplt and catpltR primers based on the pyoluteorin biosynthetic gene cluster (GenBank accession No. AY459536.1). The Pf-F and Pf-R primers (S1 Table) were designed to amplify possible homologs of a lectin-like bacteriocin gene based on the nucleotide sequence of Putidacin L1 from *P*. *protegens* CHA0 (GenBank accession No. NC_021237.1, Gene ID 57475166).

Amplification of *phlD* consisted of denaturation at 94°C for 2 min followed by 30 cycles of denaturation at 94°C for 1 min, annealing at 62°C for 45 sec, and extension at 72°C for 1 min with a final extension at 72°C for 10 min. Amplification conditions for *plt* were the same as for *phlD* with exception of extension time (2.5 min). Amplification of *llpA* consisted of denaturation at 95°C for 2 min followed by 30 cycles of denaturation at 95°C for 30 sec, annealing at 60°C for 45 sec, and extension at 72°C for 1 min with a final extension at 72°C for 5 min.

PCR amplifications were verified by agarose gel electrophoresis, and the amplified genes were purified using the QIAquick Gel Extraction Kit (Qiagen, Hilden, Germany) according to the manufacturer’s instructions. The amplified genes were sequenced by Unidad de Síntesis y Secuenciación de ADN (Instituto de Biotecnología, UNAM, México). Nucleotide sequences were analyzed using BlastN [29] against the GenBank nucleotide database.

### GenBank accession number

*P*. *protegens* EMM-1 nucleotide sequences determined herein were deposited in the GenBank database under the following accession numbers: MN959751.1 (16S rDNA), MT799749 (*rpoB*-EMM1), MT798860 (*rpoD*-EMM1), MT798861 (*gyrB*-EMM1), MT798862 (*phlD*-EMM1), MT798863 (*pltM*-EMM1), MT798859 (bacteriocin-EMM1). The sequence of the 16S rDNA gene from *Microbacterium* sp. strain UAPS01-201 was deposited in GenBank with the accession number MT095120.1.

### Antimicrobial activity assay

The ability of EMM-1 strain to inhibit the growth of microorganisms listed in Table 1 was evaluated by the double-layer agar method [1]. We used LB medium for this assay because all microbial strains used in this work were able to grow. LB contains 10 g peptone, 5 g yeast extract, and 10 g sodium chloride per liter of medium. For fungal strains, sodium chloride was removed from the LB medium (modified LB) to ensure proper growth.

For the double-layer agar method, EMM-1 was first grown in LB broth and incubated at 30°C for 24 h with reciprocal shaking (180 rpm). A 20 μL drop of the EMM-1 culture was placed in the middle of glass Petri plates containing previously poured LB agar. Plates were incubated for 48 h at 30°C. After incubation, EMM-1 colonies were removed with a sterile glass slide, and the plates were exposed to chloroform vapor for 1.5 h and left semiopen for 20 min to allow evaporation of residual chloroform. Plates were covered with 10 mL of soft agar (8 g/L) inoculated with a microbial suspension of each indicator strain, independently, and adjusted to a final concentration of 10^6^−10^8^ CFU/mL. Microbial quantification was performed by the drop-plate method [34]. For microbial suspension, bacterial colonies were grown in LB broth for 24 h at 30°C. Fungal strains were grown in modified LB broth, from the mycelium growth of a plate culture, until maximal growth (approx. 120 h).

Finally, covered plates were incubated at 30°C for 24 h (for bacteria) and 120 h (for fungi). The presence of an inhibition halo was considered indicative of the production of inhibitory substances. All assays were performed in triplicate.

### Isolation and partial characterization of the inhibitory substances

#### Cold-leaching extraction

In order to isolate the inhibitory substances produced by EMM-1, we performed the cold-leaching methodology [35]. Briefly, EMM-1 was grown on 0.22 μm nitrocellulose membranes on the surface of agar plates. Plates were incubated for 30 h at 30°C. After incubation, nitrocellulose membranes were removed, and the agar was transferred to tubes with ethanol in a ratio of 3 g/mL. Tubes were incubated at 4°C for 48 h and then centrifuged for 12 min at 4°C and 5000 rpm. The collected supernatant was concentrated on a rotary evaporator, and the aqueous phase (crude extract) was sterilized with 0.22 μm pore size filters. As a negative control, we obtained an extract from agar using the same conditions without bacterial growth.

#### Screening for antimicrobial activity of the crude extract

The antimicrobial activity of the crude extract was determined by the agar-well diffusion assay according to De Giani et al. [36] with some modifications. Petri plates were poured with soft LB agar (8 g/L) inoculated with an indicator strain. After the medium solidified, two holes (wells) were made on agar plates with 1 mL micropipette tips. Crude extract or the negative control (100 μL) was placed into each well, and the plates were incubated for 24 h at 30°C. The presence of an inhibition halo around the well was indicative of inhibitory activity. Additionally, double dilutions (1:2) of the extract were performed to determine the arbitrary units (AU/mL) of activity. One arbitrary unit is defined as the reciprocal of the highest dilution of the supernatant that inhibits the indicator strain multiplied by 1000 and divided by the volume of the extract added to the well [37, 38].

#### Molecular size evaluation and sensitivity to protease, pH and heat treatments

To determine the approximate size of the inhibitory substances, 10 mL of the crude extract was centrifuged using Millipore tubes with nitrocellulose membranes of different pore sizes (50, 30, 10, and 3 kDa) at 5000 rpm/10 min/6°C. The activity of each fraction was tested by the double-layer agar assay. For the determination of pH stability, 500 μL of the crude extract was mixed with sterile 0.01 M phosphate buffers in equal parts (1:1) and incubated for 2 h at 30°C before activity assay. Phosphate buffers were prepared using the appropriate pairs of H_3_PO_4_, NaH_2_PO_4_, and Na_2_HPO_4_ to obtain pH values ranging from 3 to 9. To test the stability of inhibitory compounds, at different temperatures, 500 μL of the crude extract was placed in Eppendorf tubes and incubated at different temperatures (ranging from -20°C to 100°C) for 80 min. Additionally, to determine the effect of proteolytic enzymes, 6 mL of the crude extract was treated with 1 mL of a *B*. *licheniformis* protease solution (P-4860, Sigma-Aldrich) at a concentration of 2.4 U/g at 30°C for 2 h. After each treatment, 300 μL of the samples were assayed for the inhibitory activity (AU/mL) against a sensitive strain (*Streptococcus* sp. SP10).

## Results

### Molecular identification of the bacterial strain EMM-1

**Fig 1:**
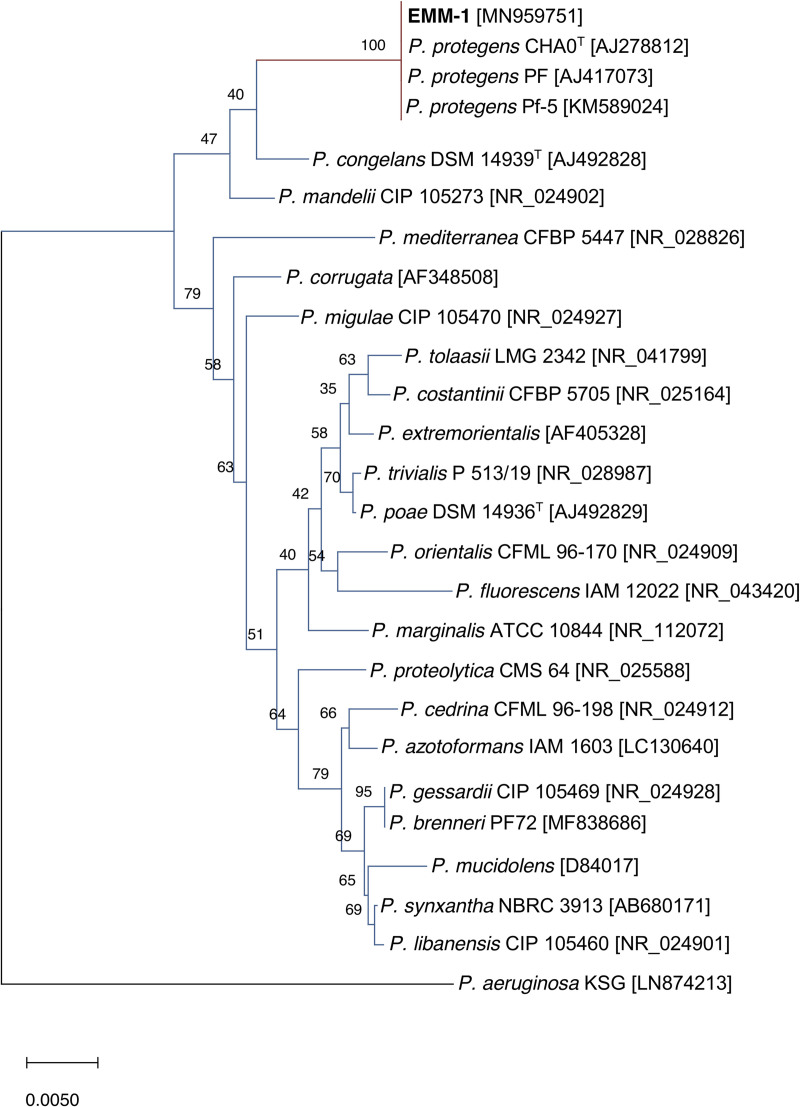
Neighbor-joining tree of partially sequenced 16S rDNA genes. The tree was designed from *P*. *fluorescens* group 16S rDNA gene sequences. GenBank accession numbers are given in brackets. The 16S rDNA gene sequence of *P*. *aeruginosa* KSG was used to root the phylogenetic tree. The scale bar indicates a genetic distance of 0.005 nt substitutions per site.

Typical bacteriological tests showed that EMM-1 strain is a Gram-negative fluorescent bacterium (S1 Fig). The bacterial genus was identified by amplifying the 16S rDNA gene using the UN27F and UN1392R primers. The obtained 16S rDNA sequence had a length of 1337 bp. Comparative analysis using BlastN [29] with whole 16S rDNA sequences suggested that the EMM-1 strain was closely related to other *Pseudomonas* species. The most closely related sequence belonged to *P*. *protegens* CHA0T (99.77% identity). Moreover,16S rDNA sequences of 24 *Pseudomonas* strains belonging to the *P*. *fluorescens* group were selected from the NCBI database to elaborate the phylogenetic tree (Fig 1). The EMM-1 strain clustered within strains of *P*. *protegens* with a bootstrap score of 100%.

**Fig 2:**
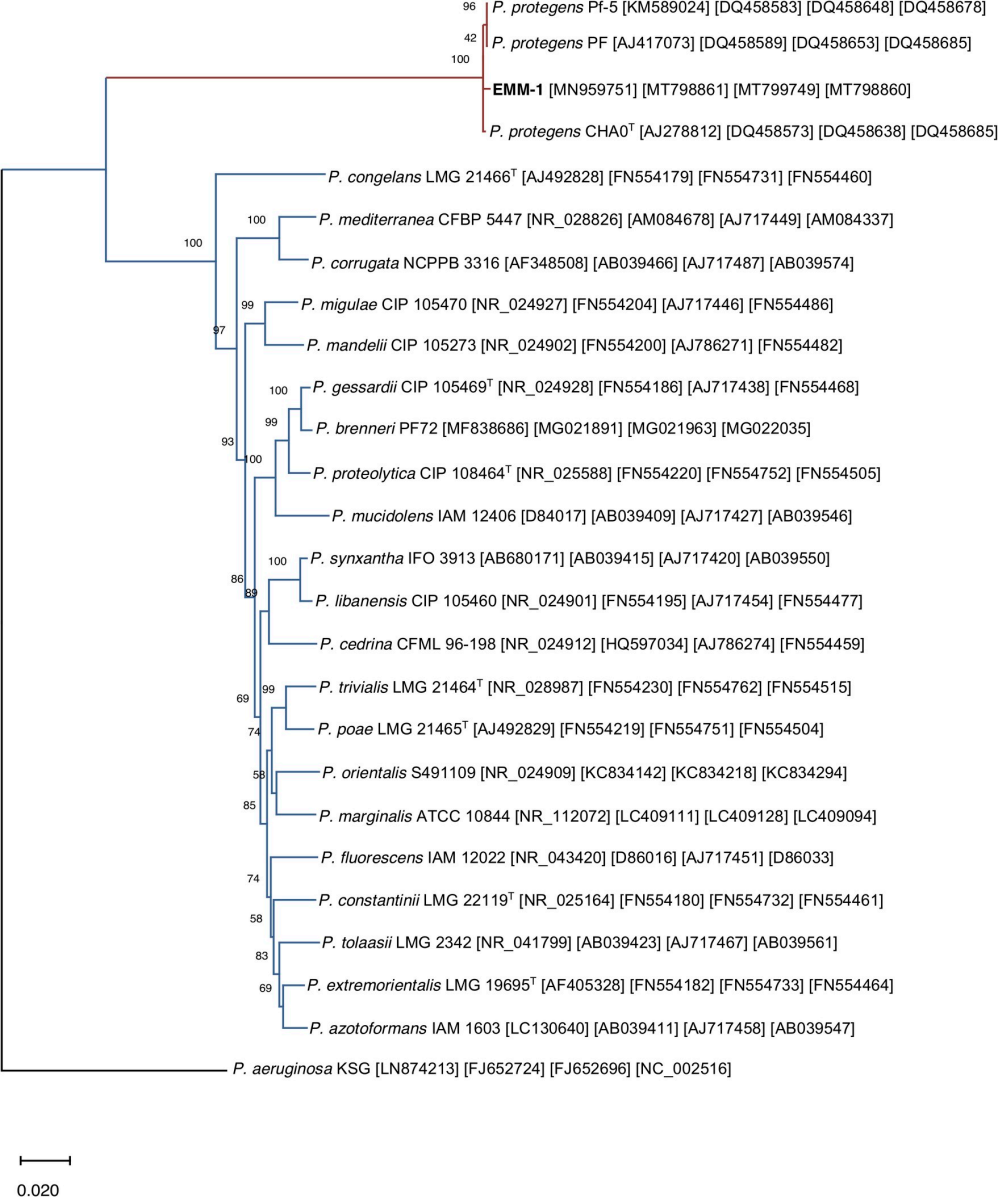
Phylogenetic tree generated by concatenation of the 16S rDNA, *rpoB*, *rpoD*, and *gyrB* gene sequences retrieved from *Pseudomonas* sp. EMM-1 and strains from *P*. *fluorescens* group. Accession numbers for 16S rDNA, *gyrB*, *rpoB* and *rpoD*, respectively, are given in brackets. *P*. *aeruginosa* KSG was used to root the phylogenetic tree. The scale bar indicates a genetic distance of 0.020 nt substitutions per site.

Because the evaluation of 16S rDNA gene sequences lacks specificity at the species level [39], three housekeeping genes (*rpoB*, *rpoD*, and *gyrB*) useful in the identification of *Pseudomonas* species were amplified for accurate identification of the EMM-1 strain. Amplification by PCR yielded amplicons of 381 bp (*rpoB*), 631 bp (*rpoD*), and 470 bp (*gyrB*) (S3 Fig). After analysis using BlastN, sequence similarity resulted in a 95–100% identity with *P*. *protegens* strains (S2 Table). Phylogenetic analysis of concatenated partial sequences of 16S rDNA, *rpoB*, *rpoD*, and *gyrB* distinguished the EMM-1 strain from other *P*. *fluorescens* strains, and EMM-1 strain clustered within *P*. *protegens* strains. This result suggests that the EMM-1 strain belongs to the *P*. *protegens* species. The clustering of *P*. *protegens* EMM-1 with other strains of the *P*. *protegens* species is shown in the respective phylogenetic tree (Fig 2).

To determine if *P*. *protegens* EMM-1 carries genes involved in the production of antimicrobials, we amplified and sequenced some genes related to the production of 2,4-DAPG, pyoluteorin, and Putidacin L1. Nucleotide sequences of 681 bp (*phlD*), 840 bp (*llpA*), and 216 bp (*pltM*) were obtained. After BlastN analysis, sequence similarity resulted in > 98% identity with sequences from *P*. *protegens* strains (S2 Table), suggesting the ability of *P*. *protegens* EMM-1 to produce these antimicrobial compounds. Further studies need to be conducted to confirming their possible role in the inhibitory activity of EMM-1 strain.

### Inhibitory activity of *P*. *protegens* EMM-1 against bacterial and fungal isolates

**Fig 3:**
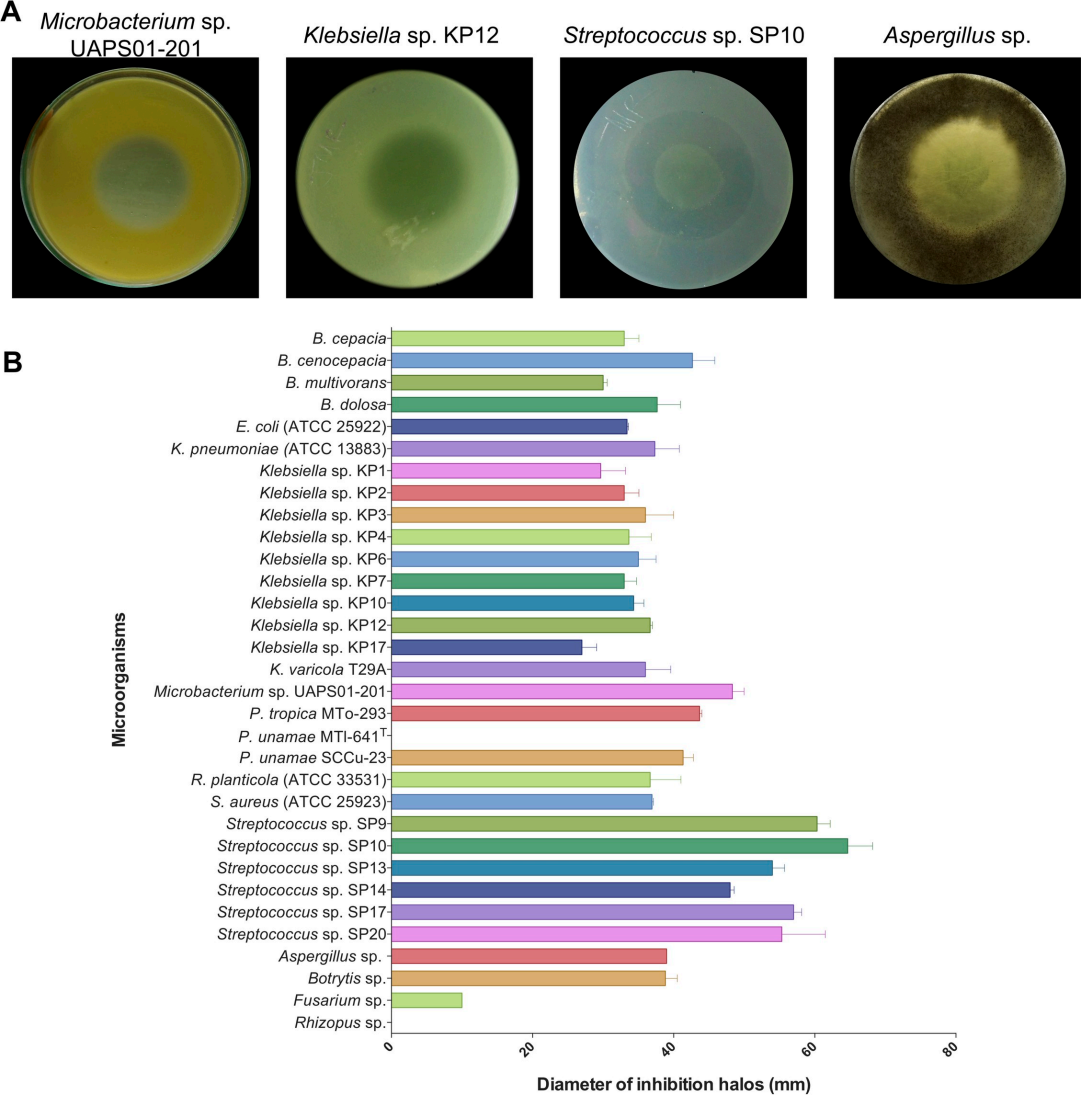
Inhibitory activity of *P*. *protegens* EMM-1 against bacterial and fungal strains. (A) Representative pictures of inhibition halos. (B) The diameter of inhibition halos (mm) was measured after 24 h of incubation for bacterial strains and after 72 h of incubation for fungal strains. Bars represent the mean of three independent replicates ± standard deviation (SD). The analysis was performed using GraphPad Prism version 6.0 software (San Diego, CA, USA).

The inhibitory activity of *P*. *protegens* EMM-1 was evaluated against 32 strains by the double-layer agar assay. All bacterial strains (except *P*. *unamae* MTl-641^T^) showed a clear inhibition halo in the middle of the plate (Fig 3A). As a result, the indicator strains were considered sensitive to the inhibitory effect of *P*. *protegens* EMM-1. After incubation of plates inoculated with *Streptococcus* strains, some colonies grew in the center of the plates, and the bacterial growth was only inhibited on the periphery, suggesting that *P*. *protegens* EMM-1 produces some inhibitory metabolites at the rim of the bacterial lawn, appearing as a ring of inhibition (Fig 3A). Gram staining was performed to investigate whether the growth in the center of the plate corresponded to the remaining cells of *P*. *protegens* EMM-1 due to improper removal of EMM-1 from the first layer (after chloroform vapors). Microscopic observation of Gram-positive cocci instead of Gram-negative *P*. *protegens* bacilli confirmed that the growth belonged to *Streptococcus* (data not shown).

The best inhibitory effect was observed for the following bacteria: *Streptococcus* sp. SP10 (64.6 ± 6.11 mm), *Microbacterium* sp. UAPS01-201 (48.3 ± 2.88 mm), *Klebsiella* sp. KP12 (36.6 ± 0.57 mm) and *P*. *tropica* MTo-293 (43.6 ± 0.57 mm) (Fig 3B and S3 Table).

Among the fungal strains evaluated, *Aspergillus* sp. and *Botrytis* sp. were considered the most sensitive with inhibition halos of 39 ± 0.00 mm and 38.8 ± 2.93 mm, respectively (Fig 3 and S3 Table). *Rhizopus* sp. was considered resistant as no inhibition halo was observed.

### Inhibitory activity of a crude extract obtained from secreted metabolites of *P*. *protegens* EMM-1

**Fig 4:**
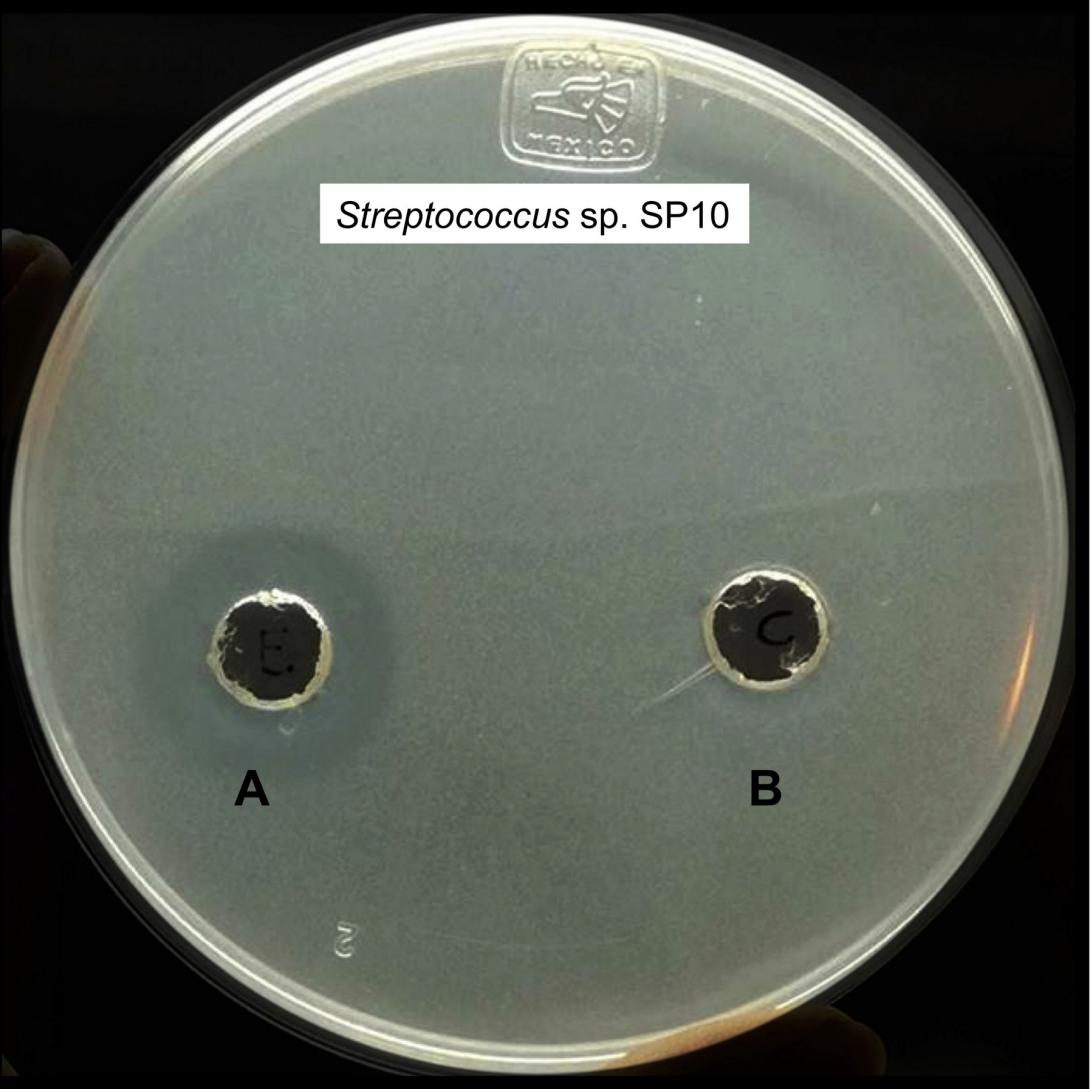
Inhibitory activity of the crude extract against *Streptococcus* sp. SP10. The crude extract (A) and a negative control (B) were evaluated by the agar-well diffusion assay.

Inhibitory substances are frequently produced and secreted by bacteria during their growth in culture media broths. To evaluate the inhibitory effect of secreted metabolites, conventional centrifugation methods for obtaining cell-free culture supernatants (CFSs) are used [40]. In the present study, CFSs from *P*. *protegens* EMM-1 did not show inhibitory activity (S2 Fig). For this reason, the inhibitory substances produced by *P*. *protegens* EMM-1 were isolated from agar by the cold-leaching methodology using ethanol. The supernatant derived from the ethanol extraction was concentrated with a rotatory evaporator, and the obtained aqueous phase was assigned as the crude extract. The inhibitory activity of the crude extract was evaluated using the agar-well diffusion assay. We selected one strain belonging to each genus of the 32 indicator strains, and the strains with the greatest inhibition halos observed in the double-layer agar assay (Fig 3B). Although *P*. *protegens* EMM-1 inhibited the growth of all bacteria tested by the double-layer agar methodology, we found that only Gram-positive strains were sensitive to the crude extract. The rest of the selected indicator strains were resistant to the concentrated crude extract, suggesting that the crude extract contains narrow-spectrum inhibitory substances directed against Gram-positive bacteria. For example, *Streptococcus* sp. SP10 was the most sensitive to the inhibitory activity of the concentrated crude extract with an inhibition halo of 17.3 ± 0.57 mm (Fig 4) and remained active until dilution 1:64. Interestingly, complete inhibition halos were observed after evaluating the crude extract against *Streptococcus* (Fig 4A) instead of the rings of inhibition formed after the double-layer agar assay (Fig 3A). *Microbacterium* sp. UAPS01-201 was only inhibited by the concentrated crude extract, with an inhibition halo of 15.6 ± 0.57 mm. Additionally, no inhibitory activity was observed against the fungal strains, indicating that antifungal substances were not present in the crude extract.

### Physicochemical characterization of partially purified inhibitory substances

To estimate the molecular size of the inhibitory substances contained in the crude extract, ultrafiltration with 50, 30, 10 and 3 kDa nitrocellulose membranes was performed, and the inhibitory activity of each fraction was evaluated using the double-layer agar assay. The fractions retained in the 50, 30, and 10 kDa membranes did not show inhibitory activity, but molecules retained in the 3 kDa filter displayed inhibitory activity, indicating an estimated molecular size between 3 to 10 kDa.

Treatment with a protease from *B*. *licheniformis* decreased the inhibitory activity of the crude extract by 90% (Table 2), suggesting that protein compounds may be present in the extract. The inhibitory activity was completely lost at 100°C but remained stable between -4°C and 60°C. Approximately 50% of the inhibitory activity remained after 80 min of incubation at -20°C and 70°C. The inhibitory activity of the crude extract was stable within the pH range of 6 to 8.

**Table 2:** Effect of pH, temperature, and enzyme treatment on the activity of the crude extract.

Treatment	Activity (AU/mL)*
**Enzyme**	
Protease from *B*. *licheniformis*	1
Temperature (°C)	
-20	4
-4	8
4	8
30	8
60	8
70	4
80	2
100	0
**pH**	
3	4
4	4
5	4
6	8
7	8
8	8
9	4

*Inhibitory activity was tested against the SP10 strain in each treatment. The divisible values of AU/mL obtained are represented in this table.

## Discussion

*Pseudomonas* are ubiquitous Gram-negative bacteria highly competitive in bacterial communities [41]. The organisms of the *Pseudomonas* genus produce an arsenal of inhibitory substances like broad-spectrum antibiotics, siderophores, lytic enzymes, bacteriocins, and antifungal compounds [7, 42]. *P*. *protegens* is a plant-protecting bacterium that produces pyoluteorin and 2,4-diacetylphloroglucinol (2,4-DAPG) antimicrobial compounds. This bacterium has been studied for its biocontrol properties because it inhibits several phytopathogenic fungi. However, little is known about the antibacterial activity of *P*. *protegens* against bacterial clinical isolates [6].

In this study, a new antagonistic bacterium isolated from the rhizosphere of “Rojo Criollo” maize, EMM-1, was assigned to the *P*. *protegens* species based on the phylogenetic analysis of 16S rDNA, *rpoB*, *rpoD*, and *gyrB* gene sequences (Fig 2). This strain also harbored genes involved in the synthesis of 2,4-DAPG (*phlD*) and pyoluteorin (*pltM*) (S3 Fig). Therefore, it can be inferred that *P*. *protegens* EMM-1 produces relevant antifungal metabolites, which is consistent with the inhibitory effect displayed against the fungal strains evaluated in the double-layer agar assay. Previous studies have demonstrated the potential of *P*. *protegens* to inhibit fungal pathogens, mainly involved in plant diseases, including *Botrytis*, *Aspergillus*, and *Fusarium* [6], which were all considered in this study. *P*. *protegens* EMM-1 showed inhibitory activity against those important fungi (Fig 3), but no inhibitory activity was displayed against *Rhizopus* sp. The production of lectin-like bacteriocins (LlpAs) in *P*. *protegens* has been well documented. LlpAs are 31-kDa proteins composed of two monocot mannose-binding lectin domains [2]. Here, we showed that *P*. *protegens* EMM-1 carries a gene involved in the synthesis of a lectin-like bacteriocin of the Putidacin L1 family from *P*. *protegens* CHA0 (S3 Fig). Understanding which of these antimicrobials, 2,4-DAPG, pyoluteorin, or Putidacin L1, may be responsible for the inhibitory effect of *P*. *protegens* EMM-1, will be of interest to study.

The inhibitory potential of *P*. *protegens* against bacterial strains such as *Pseudomonas putida*, *Pseudomonas syringae*, *Ralstonia solanacearum*, *Enterobacter* sp., and *Bacillus subtilis* has been demonstrated [43–45]. However, to our knowledge, the antibacterial activity of *P*. *protegens* against MDR clinical isolates has not been reported. In this study, the inhibitory potential of *P*. *protegens* EMM-1 against clinical isolates was revealed. *P*. *protegens* EMM-1 inhibited Gram-positive and Gram-negative bacteria, including MDR opportunistic pathogens, through the production of inhibitory substances (Fig 3 and S3 Table). The strongest inhibition was observed against β-hemolytic *Streptococcus*. Members of the *B*. *cepacia* complex, *R*. *planticola*, and *K*. *pneumoniae* were also inhibited. Although the inhibitory potential of some fluorescent pseudomonads against clinical isolates, such as *S*. *aureus*, has been demonstrated [46], little is known about the inhibitory activity against the MDR strains evaluated in this work.

Besides, *P*. *protegens* EMM-1 inhibited diverse rhizospheric bacteria (Fig 3B and S3 Table). When an antagonistic bacterium is used for the biocontrol of phytopathogens, its effectiveness depends on the ability to colonize the surface or roots of the plants and quickly consume the nutrients that are also essential for the pathogens. It has been established that the production of inhibitory compounds provides these competitive advantages to antagonistic bacteria [47]. In this work, evaluating the inhibitory activity of *P*. *protegens* EMM-1 against rhizospheric strains allowed us to know its competition potential, since it inhibited the growth of most of the rhizospheric strains evaluated. Although the inhibition potential of a microorganism *in vitro* does not guaranty its inhibitory activity *in situ*, these attributes may provide *P*. *protegens* EMM-1 competitive advantages to colonize diverse environments.

Some bacteria capable of producing inhibitory substances have been used for plant growth promotion and are marketed as mono or multi-inoculants. These bacteria have been found to be beneficial for the biocontrol of plant pathogens [48–50]. However, many plant growth-promoting rhizobacteria (PGPR) have failed as bioinoculants because of their lack of competitiveness, or because there exists some inhibition between them [51]. Therefore, our results provide a key finding if future formulations of *P*. *protegens* EMM-1 are intended to be formulated in co-inoculation with other PGPR.

Inhibitory substances produced by bacteria can be obtained and purified by different methodologies, including ammonium sulfate precipitation, solvent extraction, inactivation of bacterial cultures by heat [40, 52], or by preparation of CFSs from liquid cultures [53]. In the present study, a free-cell supernatant from *P*. *protegens* EMM-1 did not show inhibitory activity (S2 Fig), but a crude extract obtained from agar did show activity. The crude extract displayed inhibitory activity against *Streptococcus* sp. SP10 (Fig 4) and *Microbacterium* sp. UAPS01-201. However, the crude extract did not show an inhibitory effect against fungi, suggesting that the isolated inhibitory substances have a narrow inhibitory spectrum only against Gram-positive bacteria.

The physicochemical characterization of partially purified inhibitory substances produced by *P*. *protegens* EMM-1 revealed an estimated molecular mass of 3 to 10 kDa. Although we cannot exclude the possibility that other inhibitory substances can be obtained by other methodologies as reported in other *Pseudomonas* species [54, 55]. The inhibitory activity remained active at low pH and was not affected by heat treatment. However, the inhibitory activity was lost at 100°C. Moreover, the inhibitory activity decreased by 90% when treated with a protease, suggesting the presence of protein compounds in the crude extract. These characteristics are commonly related to some bacteriocins produced by *Pseudomonas* strains [56]. Additional studies are required to confirm the chemical nature of the inhibitory substances isolated in this work.

Currently, there is an urgent need to discover new antimicrobials with new mechanisms of action because the increase of MDR pathogens is becoming a public health problem [14]. In fact, for every new antimicrobial that is discovered, bacteria develop the ability to defeat them, either eliminating or reducing their effectiveness [57]. Thus, the use of inhibitory substances produced by antagonistic bacteria may complement conventional antimicrobial therapies [58]. For several years, inhibitory substances as bacteriocins have been approved as preservatives for diverse food products, and their efficacy to decrease the number of pathogens *in vivo* models has been evaluated [11, 12]. Bacteriocins from *P*. *aeruginosa*, *E*. *coli*, and *Planomonospora* sp. have demonstrated to be more effective than other approved antibiotics for the treatment of MDR pathogens [59]. Although our study did not evaluate pure compounds, the use of semipurified crude extract or the producer strain as a probiotic agent could be applied in food [10]. Further studies should be performed *in vivo* to evaluate the potential use of the crude extract or EMM-1 strain for these purposes.

Our results demonstrated the potential of *P*. *protegens* EMM-1 as a possible inhibitor of MDR clinical pathogens and phytopathogenic fungi. The use of *P*. *protegens* EMM-1 as bioinoculant may be beneficial for biocontrol of plant or postharvest diseases as well as a possible probiotic bacterium.

## Conclusions

A new antagonistic bacterium isolated from the rhizosphere of “Rojo Criollo” maize, was identified as *P*. *protegens* EMM-1 after phylogenetic analysis. This investigation showed that *P*. *protegens* EMM-1 can efficiently inhibit the growth of a broad spectrum of bacteria, including MDR clinical isolates *Klebsiella* sp. and *Streptococcus* sp. *P*. *protegens* EMM-1 also inhibited to some important fungi such as *Botrytis* sp. and *Aspergillus* sp. Therefore, the ability of *P*. *protegens* EMM-1 to inhibit these microorganisms should be tested *in vivo* to determine its probiotic or biocontrol agent potential.

Moreover, some inhibitory substances produced by *P*. *protegens* EMM-1 in solid media and extracted by the cold-leaching methodology displayed strong inhibitory activity against Gram-positive bacteria, which suggests the existence of other inhibitory substances that could be explored in the future.

This work demonstrated the significance of evaluating rhizospheric bacteria to discover inhibitory compounds that may potentially be used for the development of new antimicrobial therapies for application in medicine or agriculture. Furthermore, our findings encourage the design of new methodologies to recover unexplored inhibitory substances.

## Supporting information

S1 Fig Fluorescence observed under UV light. (PDF)

S2 Fig Inhibitory activity assay of a cell-free supernatant obtained from a liquid culture of *P*. *protegens* EMM-1. (PDF)

S3 Fig Agarose gel electrophoresis of the PCR products amplified from the genomic DNA of EMM-1 strain. (PDF)

S1 Table Primers used for PCR amplification. (PDF)

S2 Table BLAST analysis of the nucleotide sequences amplified. (PDF)

S3 Table Inhibitory activity of *P*. *protegens* EMM-1 in the double-layer agar assay. (PDF)

S1 Raw image Agarose gel electrophoresis of PCR amplified products from the genomic DNA of the EMM-1 strain. Lane 1: 1 kb Plus DNA Ladder (Thermo Scientific™, Carlsbad, CA, USA); 2. 16S rDNA; 3. *rpoB*; 4. *rpoD*; 5. *gyrB*; 6. *phlD*; 7. *plt*; 8. *llpA*. The PCR products were electrophoresed on a 1% agarose gel (50 min/90 volts) and visualized under UV light in a Spectroline Ultraviolet Transilluminator, using GelRed^®^ Nucleic Acid Gel Stain (Biotium, Inc., Fremont, CA, USA). Nonspecific bands are observed in lanes 2, 3, and 8. The photography was captured with a Huawei P9 Lite VNS-L53 camera and 1–8 lanes were selected to generate the S3 Fig.
(PDF)
