# Supplementary material for: Growth inhibition of pathogenic microorganisms by Pseudomonas protegens EMM-1 and partial characterization of inhibitory substances
Source: PLoS One. 2020 Oct 15;15(10):e0240545. doi: 10.1371/journal.pone.0240545 (PMC7561207; doi:10.1371/journal.pone.0240545)
Supplement: S1 Fig — (PDF) [file pone.0240545.s001.pdf]

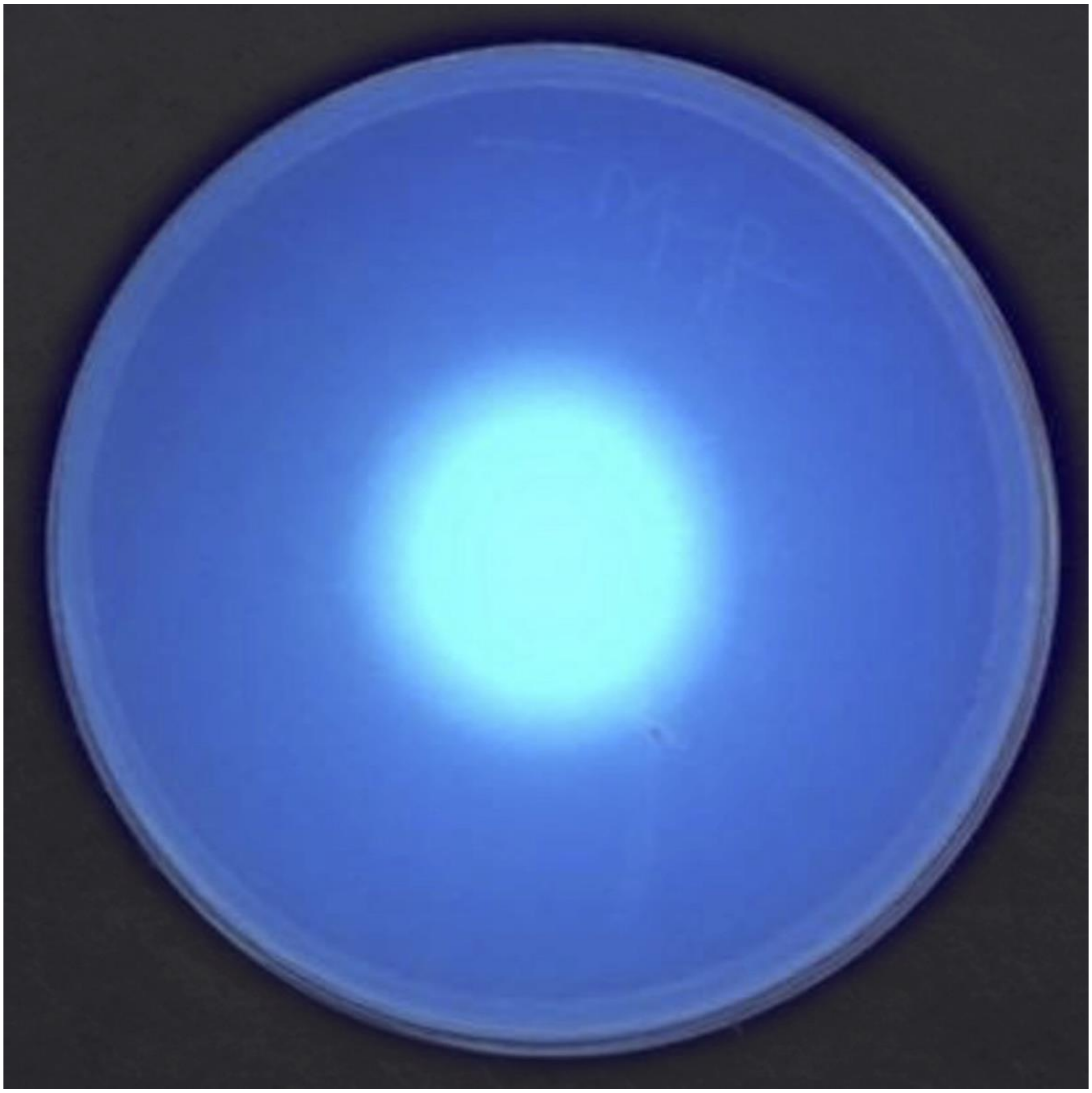

S1 Fig. Fluorescence observed under UV light. *P. protegens* EMM-1 was growth in *Pseudomonas* isolation agar which enhance the formation of blue or blue-green pyocyanin pigments by *Pseudomonas* species.
