# Supplementary material for: Growth inhibition of pathogenic microorganisms by Pseudomonas protegens EMM-1 and partial characterization of inhibitory substances
Source: PLoS One. 2020 Oct 15;15(10):e0240545. doi: 10.1371/journal.pone.0240545 (PMC7561207; doi:10.1371/journal.pone.0240545)
Supplement: S2 Fig — (PDF) [file pone.0240545.s002.pdf]

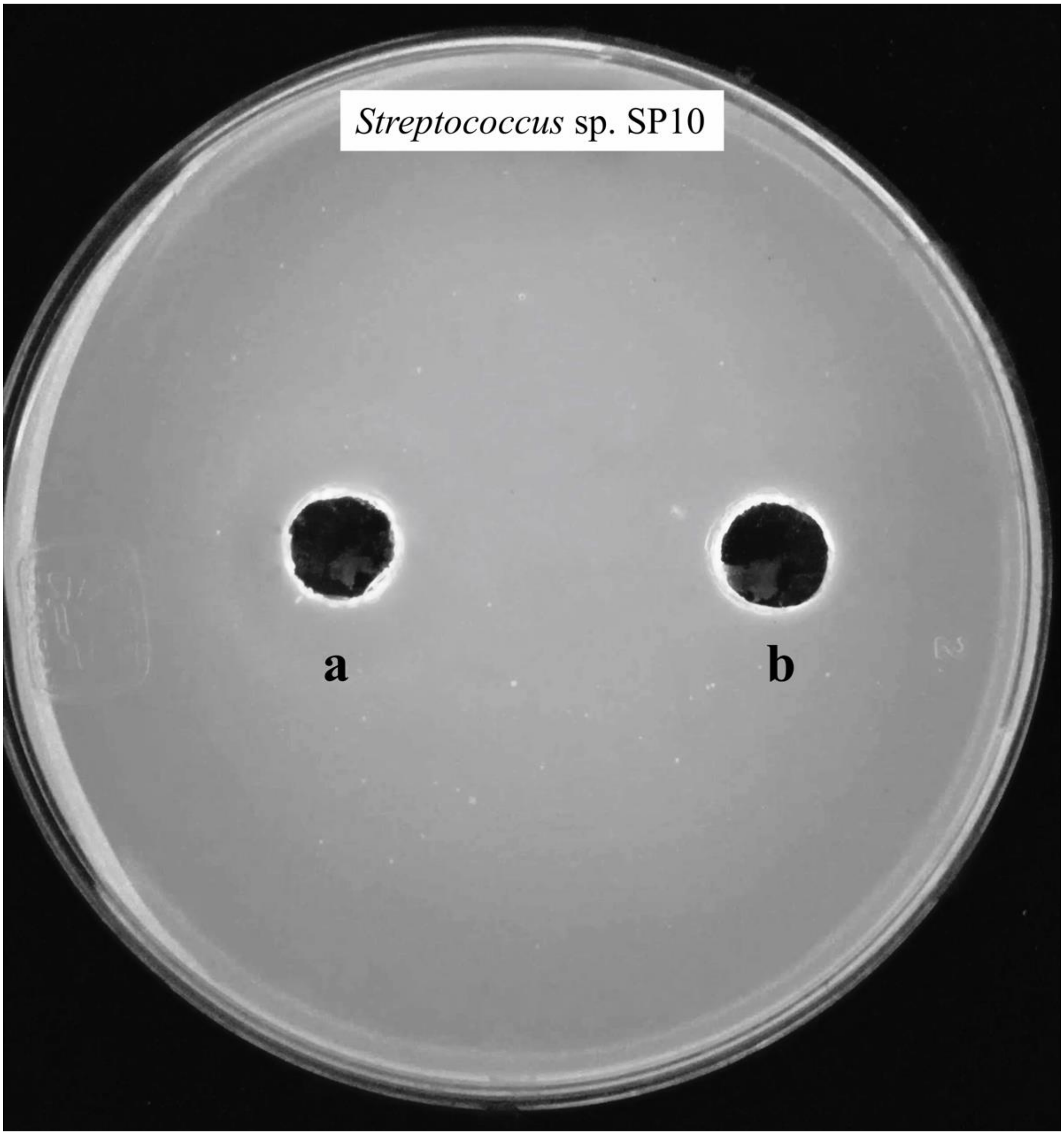

S2 Fig. Inhibitory activity assay of a cell-free supernatant obtained from a liquid culture of *P. protegens* EMM-1. The free-cell supernatant (a) and a negative control (sterile LB) (b) were evaluated by the agar-well diffusion assay against *Streptococcus* sp. SP10. No inhibition halo was observed after incubation of 24 h.
