# Supplementary material for: Growth inhibition of pathogenic microorganisms by Pseudomonas protegens EMM-1 and partial characterization of inhibitory substances
Source: PLoS One. 2020 Oct 15;15(10):e0240545. doi: 10.1371/journal.pone.0240545 (PMC7561207; doi:10.1371/journal.pone.0240545)
Supplement: S3 Fig — (PDF) [file pone.0240545.s003.pdf]

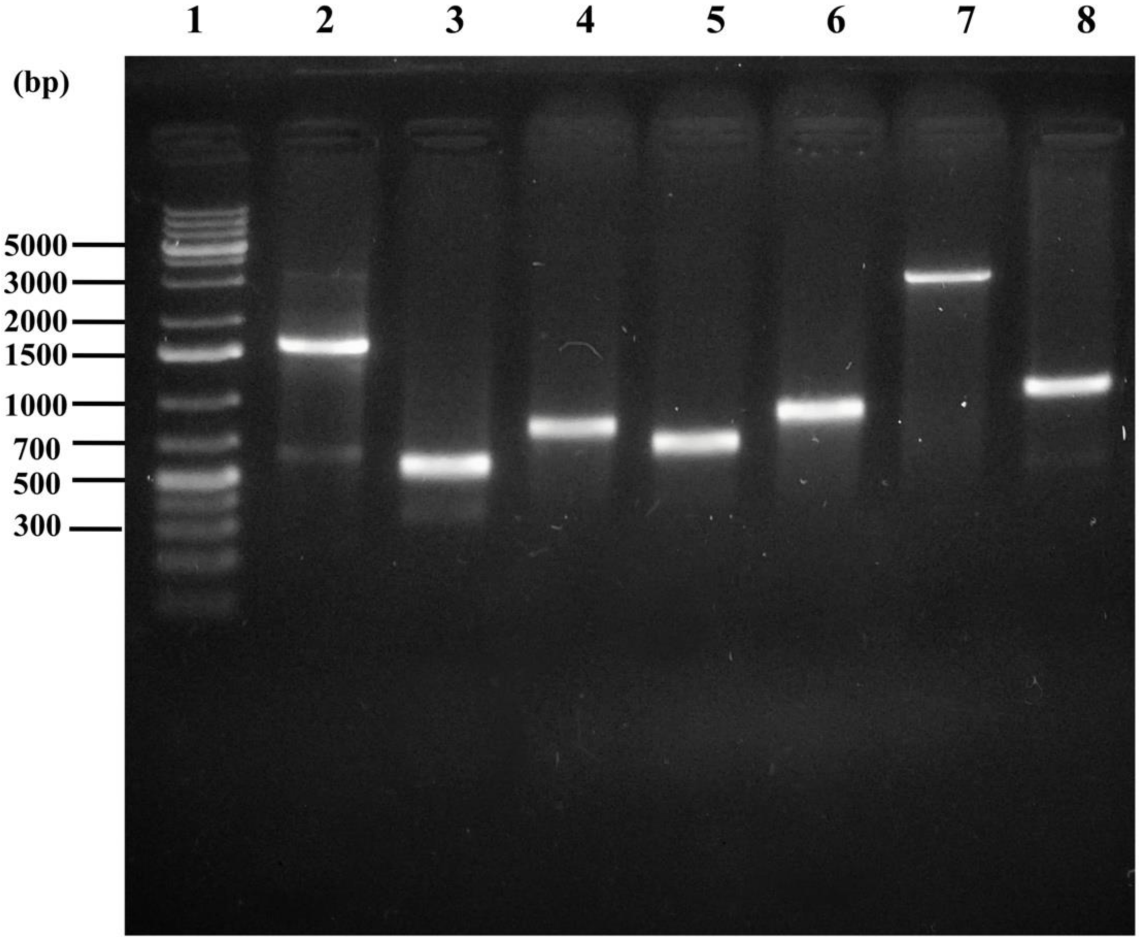

S3 Fig. Agarose gel electrophoresis of the PCR products amplified from the genomic DNA of EMM-1 strain. Lane 1: 1 kb Plus DNA ladder (Thermo Scientific™); 2. 16S rDNA; 3. *rpoB*; 4. *rpoD*; 5. *gyrB*; 6. *phlD*; 7. *plt*; 8. *llpA*. Non-specific bands are observed in lanes 2, 3, and 8.
