# Supplementary material for: Growth inhibition of pathogenic microorganisms by Pseudomonas protegens EMM-1 and partial characterization of inhibitory substances
Source: PLoS One. 2020 Oct 15;15(10):e0240545. doi: 10.1371/journal.pone.0240545 (PMC7561207; doi:10.1371/journal.pone.0240545)
Supplement: S1 Table — (PDF) [file pone.0240545.s004.pdf]

S1 Table. Primers used for PCR amplification

Gene	Product	Size (~bp)	Forward (F) and reverse (R) primers (5' → 3')	Tm (ºC)	Reference
rrs	16S rDNA	1500	UN27F (F): TAGAGTTTGATCCTGGCTCAG<br>UN1392R (R): CAGGGGCGGTGTGTACA	52	[1]
rpoB	RNA polymerase β-subunit	508	rpoBf1 (F): CAGTTCATGGACCAGAACAACCCGCT<br>rpoBr1 (R): CCCATCAACGCACGGTTGGCGTC	60	[2]
rpoD	RNA polymerase σ70 factor	695	rpoDf (F): ACTTCCCTGGCACGGTTGACCA<br>rpoDr (R): TCGACATGCGACGGTTGATGTC	60	[2]
gyrB	DNA gyrase β-subunit	586	gyrBf (F): TTCAGCTGGGACATCCTGGCCAA<br>gyrBr2 (R): TCGATCATCTTGCCGACRACCA	65	[2]
phlD	Polyketide synthase (PKS)	745	Phl2a (F): GAGGACGTCGAAGACCACCA<br>Phl2b (R): ACCGCAGCATCGTGTATGAG	62	[3]
plt	Pyoluteorin biosynthetic gene cluster	2444	catplt (F): GCAGCAGCGTCAATGATGAAC<br>catpltR (R): CATGCTCGCGCTCGAACAGTTC	62	This work
llpA	Lectin-like bacteriocin	843	Pf-F (F): ATGGCTTGGATTCGATACGAC<br>Pf-R (R): TTAGAACACGTTGTCCCAGGT	60	This work

S1 Table References.

1. Morales-García YE, Juárez-Hernández D, Aragón-Hernández C, Mascarua-Esparza MA, Bustillos-Cristales MR, Fuentes-Ramírez LE, et al. Growth response of maize plantlets inoculated with *Enterobacter* spp., as a model for alternative agriculture. Rev Argent Microbiol. 2011;43: 287–293. doi:10.1590/S0325-75412011000400009

2. Frapolli M, Défago G, Moënne-Loccoz Y. Multilocus sequence analysis of biocontrol fluorescent *Pseudomonas* spp. producing the antifungal compound 2,4-diacetylphloroglucinol. Environ Microbiol. 2007;9: 1939–1955. doi:10.1111/j.1462-2920.2007.01310.x

3. Ramette A, Moënne-Loccoz Y, Défago G. Polymorphism of the polyketide synthase gene phlD in biocontrol fluorescent pseudomonads producing 2,4-diacetylphloroglucinol and comparison of PhlD with plant polyketide synthases. Mol PlantMicrobe Interact. 2001;14: 639–652. doi:10.1094/MPMI.2001.14.5.639
