# Supplementary material for: Growth inhibition of pathogenic microorganisms by Pseudomonas protegens EMM-1 and partial characterization of inhibitory substances
Source: PLoS One. 2020 Oct 15;15(10):e0240545. doi: 10.1371/journal.pone.0240545 (PMC7561207; doi:10.1371/journal.pone.0240545)
Supplement: S2 Table — (PDF) [file pone.0240545.s005.pdf]

S2 Table. BLAST analysis of the nucleotide sequences amplified.

Gene	Closest relative microorganism	Identity (%)	GenBank accession number
1. 16S rDNA	*P. protegens* strain CHA0T	99.77	AJ278812.1
	P. protegens strain PF	99.77	AJ417073.1
	P. protegens strain H407	99.77	MH669312.1
	P. protegens strain E2HL9	99.77	MK855122.1
	P. protegens Pf-5	99.77	CP000076.1
2. *rpoB*	*Pseudomonas* sp. MP12	95.43	KX236070.1
	P. protegens strain UCT	95.20	CP017964.1
	P. protegens strain pf5-k3	95.16	CP032352.1
	P. protegens strain FDAARGOS_307	95.16	CP022097.2
	P. protegens strain 14B2	95.16	KX696870.1
3. *rpoD*	*Pseudomonas* sp. JV391_D9	99.83	LN885701.1
	P. protegens strain BR3c	99.68	MH185796.1
	Pseudomonas sp. RW09-C30	99.66	JN397603.1
	Pseudomonas sp. Irchel 2B15	99.52	LS399164.1
	Pseudomonas sp. strain S3Bt35	99.51	MH494138.1
4. *gyrB*	*P. fluorescens* strain PGNL1	100.00	FJ012221.1
	P. protegens strain 1B1	99.58	KX696641.1
	P. protegens strain H78	99.58	CP013184.1
	P. protegens strain UCT	99.58	CP017964.1
	P. protegens strain Pf2	99.58	KU052582.1
5. *phlD*	*P. protegens* Cab57	99.41	AP014522.1
	P. protegens strain pf5-k2	98.97	CP032353.1
	P. protegens CHA0	98.97	LS999205.1
	P. protegens strain FDAARGOS_307	98.97	CP022097.2
	P. protegens strain H78	98.97	CP013184.1
6. *plt*	*P. protegens* Cab57	100.00	AP014522.1
	P. protegens CHA0	100.00	CP003190.1
	P. fluorescens strain Pf-5	100.00	AF081920.3
	P. protegens CHA0	100.00	AY459536.1
	P. protegens strain FD6	99.29	CP031396.1
7. *llpA*	*P. protegens* strain SN15-2	99.76	CP043179.1
	P. protegens strain pf5	99.76	CP032358.1
	P. protegens CHA0	99.76	LS999205.1
	P. protegens strain FDAARGOS_307	99.76	CP022097.2
	P. protegens strain H78	99.76	CP013184.1
