# Supplementary material for: Growth inhibition of pathogenic microorganisms by Pseudomonas protegens EMM-1 and partial characterization of inhibitory substances
Source: PLoS One. 2020 Oct 15;15(10):e0240545. doi: 10.1371/journal.pone.0240545 (PMC7561207; doi:10.1371/journal.pone.0240545)
Supplement: S3 Table — (PDF) [file pone.0240545.s006.pdf]

S3 Table. Inhibitory activity of *P. protegens* EMM-1 in the double-layer agar assay. Diameter of inhibition halos (mm) was measured after 24 h of incubation for bacterial strains, and after 120 h of incubation for fungal strains. Bars represent the mean of three independent replicates ± standard deviation.

**Microorganism**	**Diameter of inhibition halos (mm ± SD[1])**
1. *B. cepacia*	33.0 ± 3.60
2. *B. cenocepacia*	42.6 ± 5.50
3. *B. multivorans*	30.0 ± 1.00
4. *B. dolosa*	37.6 ± 5.68
5. *E. coli* ATCC 25922	33.4 ± 0.34
6. *K. pneumoniae* subsp. *pneumoniae* ATCC 13883	37.3 ± 6.02
7. *Klebsiella* sp. KP1	29.6 ± 6.11
8. *Klebsiella* sp. KP2	33.0 ± 3.60
9. *Klebsiella* sp. KP3	36.0 ± 6.92
10. *Klebsiella* sp. KP4	33.6 ± 5.50
11. *Klebsiella* sp. KP6	35.0 ± 4.35
12. *Klebsiella* sp. KP7	33.0 ± 3.00
13. *Klebsiella* sp. KP10	34.3 ± 2.51
14. *Klebsiella* sp. KP12	36.6 ± 0.57
15. *Klebsiella* sp. KP17	27.0 ± 3.60
16. *K. variicola* T29A	36.0 ± 6.24
17. *Microbacterium* sp. UAPS01-201	48.3 ± 2.88
18. *P. tropica* MTo-293	43.6 ± 0.57
19. *P. unamae* MTl-641^T	Non detected (ND)
20. *P. unamae* SCCu-23	41.3 ± 2.51
21. *R. planticola* ATCC 33531	36.6 ± 7.57
22. *S. aureus* subsp. *aureus* ATCC 25923	36.9 ± 0.37
23. *Streptococcus* sp. SP9	60.3 ± 3.21
24. *Streptococcus* sp. SP10	64.6 ± 6.11
25. *Streptococcus* sp. SP13	54.0 ± 3.00
26. *Streptococcus* sp. SP14	48.0 ± 1.00
27. *Streptococcus* sp. SP17	57.0 ± 2.00
28. *Streptococcus* sp. SP20	55.3 ± 10.6
29. *Aspergillus* sp.	39.0 ± 0.00

30. *Botrytis* sp.	38.8 ± 2.93
31. *Fusarium* sp.	10.0 ± 0.00
32. *Rhizopus* sp.	ND

[1]Values represent the mean of three independent determinations ± standard deviation (SD).
