# Supplementary material for: Growth inhibition of pathogenic microorganisms by Pseudomonas protegens EMM-1 and partial characterization of inhibitory substances
Source: PLoS One. 2020 Oct 15;15(10):e0240545. doi: 10.1371/journal.pone.0240545 (PMC7561207; doi:10.1371/journal.pone.0240545)
Supplement: S1 Raw image — Lane 1: 1 kb Plus DNA Ladder (Thermo Scientific™, Carlsbad, CA, USA); 2. 16S rDNA; 3. rpoB; 4. rpoD; 5. gyrB; 6. phlD; 7. plt; 8. llpA. The PCR products were electrophoresed on a 1% agarose gel (50 min/90 volts) and visualized under UV light in a Spectroline Ultraviolet Transilluminator, using GelRed® Nucleic Acid Gel Stain (Biotium, Inc., Fremont, CA, USA). Nonspecific bands are observed in lanes 2, 3, and 8. The photography was captured with a Huawei P9 Lite VNS-L53 camera and 1–8 lanes were selected to generate the S3 Fig. (PDF) [file pone.0240545.s007.pdf]

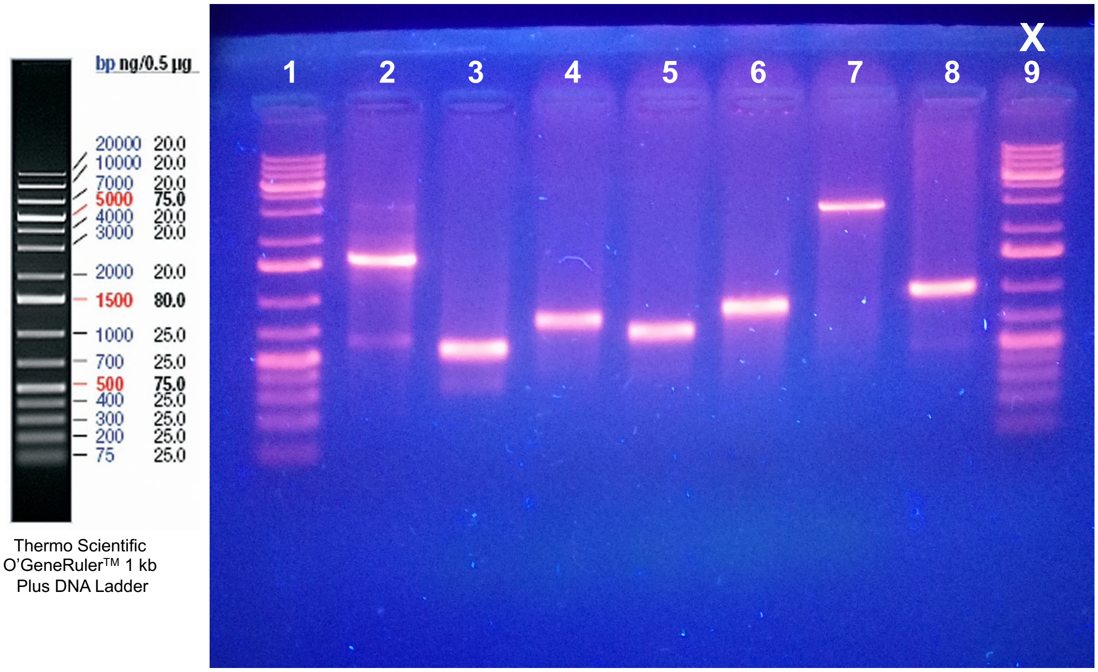

S1 raw image. Agarose gel electrophoresis of PCR amplified products from the genomic DNA of the EMM-1 strain. Lane 1: 1 kb Plus DNA Ladder (Thermo Scientific™, Carlsbad, CA, USA); 2. 16S rDNA; 3. *rpoB*; 4. *rpoD*; 5. *gyrB*; 6. *phlD*; 7. *plt*; 8. *llpA*. The PCR products were electrophoresed on a 1 % agarose gel (50 min/90 volts) and visualized under UV light in a Spectroline Ultraviolet Transilluminator, using GelRed® Nucleic Acid Gel Stain (Biotium, Inc., Fremont, CA, USA). Nonspecific bands are observed in lanes 2, 3, and 8. The photography was captured with a Huawei P9 Lite VNS-L53 camera and 1-8 lanes were selected to generate the S3 figure.
